# Effect of Butyrate on Food-Grade Titanium Dioxide Toxicity in Different Intestinal In Vitro Models

**DOI:** 10.1021/acs.chemrestox.4c00086

**Published:** 2024-08-30

**Authors:** Janine M. Becht, Hendrik Kohlleppel, Roel P. F. Schins, Angela A. M. Kämpfer

**Affiliations:** IUF—Leibniz Research Institute for Environmental Medicine, Düsseldorf 40225, Germany

## Abstract

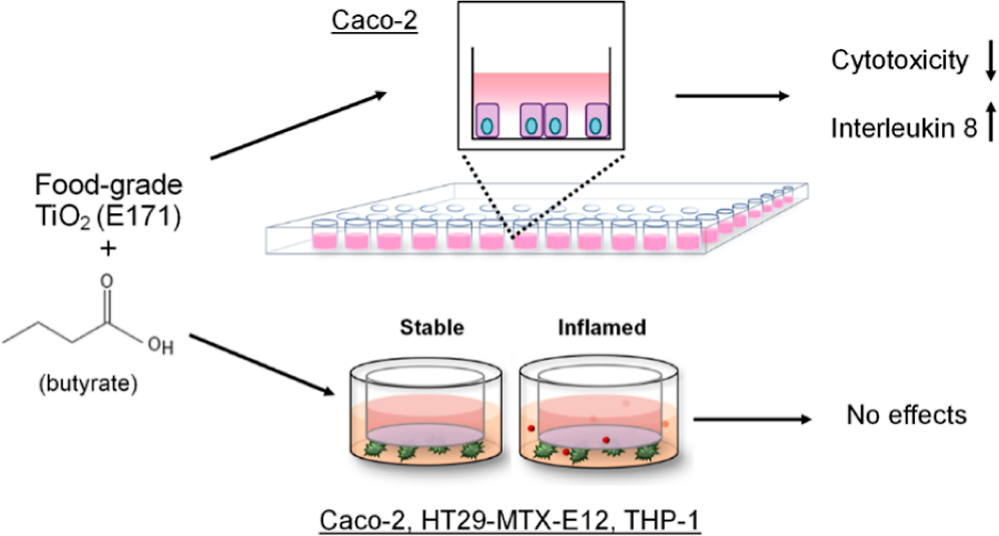

Short-chain fatty acids (SCFA) are an important energy source for colonocytes and crucial messenger molecules both locally in the intestine and systemically. Butyrate, one of the most prominent and best-studied SCFA, was demonstrated to exert anti-inflammatory effects, improve barrier integrity, enhance mucus synthesis in the intestine, and promote cell differentiation of intestinal epithelial cells in vitro. While the physiological relevance is undisputed, it remains unclear if and to what extent butyrate can influence the effects of xenobiotics, such as food-grade titanium dioxide (E171, _fg_TiO_2_), in the intestine. TiO_2_ has been controversially discussed for its DNA-damaging potential and banned as a food additive within the European Union (EU) since 2022. First, we used enterocyte Caco-2 monocultures to test if butyrate affects the cytotoxicity and inflammatory potential of _fg_TiO_2_ in a pristine state or following pretreatment under simulated gastric and intestinal pH conditions. We then investigated pretreated _fg_TiO_2_ in intestinal triple cultures of Caco-2, HT29-MTX-E12, and THP-1 cells in homeostatic and inflamed-like state for cytotoxicity, barrier integrity, cytokine release as well as gene expression of mucins, oxidative stress markers, and DNA repair. In Caco-2 monocultures, butyrate had an ambivalent role: pretreated but not pristine _fg_TiO_2_ induced cytotoxicity in Caco-2 cells, which was not observed in the presence of butyrate. Conversely, _fg_TiO_2_ induced the release of interleukin 8 in the presence but not in the absence of butyrate. In the advanced in vitro models, butyrate did not affect the characteristics of the healthy or inflamed states and caused negligible effects in the investigated end points following _fg_TiO_2_ exposure. Taken together, the effects of _fg_TiO_2_ strongly depend on the applied testing approach. Our findings underline the importance of the experimental setup, including the choice of in vitro model and the physiological relevance of the exposure scenario, for the hazard testing of food-grade pigments like TiO_2_.

## 1 Introduction

Short-chain fatty acids (SCFA) are byproducts of intestinal bacterial fermentation, mainly produced by *Firmicutes* species of nondigestible dietary fibers.^1^ They are crucial for intestinal development and maintenance of homeostasis in the gut (reviewed by Parada Venegas et al.^1^ and Chen and Vitetta^2^) and serve regulatory functions on extra-intestinal organs.^3^ Accounting for ∼20%, butyrate is one of the most abundant SCFA. As an important energy source, it is rapidly metabolized by colonocytes under oxygen consumption, thereby supporting the establishment of the oxygen gradient and maintenance of anaerobic conditions in the intestine.^4^ Butyrate affects intestinal epithelial cell differentiation by influencing the cell cycle, enhancing barrier integrity, and brush border enzymes like alkaline phosphatase.^5−8^ Due to its effects on cell cycle arrest, apoptosis, and inhibition of cell migration, butyrate is suggested to protect against malignant developments.^9−12^

Considering its physiological importance, it does not come as a surprise that butyrate dysregulation is prevalent in numerous intestinal diseases and disorders. In inflammatory bowel diseases, a shift in the microbial composition, reduction in butyrate-producing species, and overall lower levels of SCFA are prevalent.^13−15^ In this context, in vivo studies demonstrated a protective effect of butyrate preincubation and, to a lesser extent, butyrate coexposure in dextran sulfate sodium and 2,4,6-trinitrobenzenesulfonic acid induced colitis mouse models. Butyrate-treated mice displayed better clinical and histological scores than the control group, less inflammatory cell infiltration, and overall preserved epithelial and mucus barriers.^16,17^

In vitro studies have mainly been used to unravel the mechanisms of butyrate-induced effects. Butyrate was found to be involved in the rescue of cellular energy metabolism while improving mitochondrial function during physiological stress,^18^ to exert overall cytoprotective effects against reactive oxygen species (ROS)-induced damage,^19^ and to protect intestinal epithelial cells from pathogenic invasion.^20^ Its immunomodulatory function was demonstrated in both epithelial and immune cells where butyrate modulates the release of pro-inflammatory mediators in response to microbial stressors.^21−24^

In a recent study exposing mice to different types of engineered nanomaterials (ENM) in feed pellets, we have identified an increased presence of the butyrate-producing genus *Roseburia* following a 28 day oral exposure to silver ENM.^25^ Together with the mentioned cytoprotective effects of butyrate, this finding sparked our interest to investigate whether butyrate affects the toxicity of xenobiotics in intestinal in vitro systems, which to our knowledge, has not been investigated yet. The study was conducted with food-grade titanium dioxide (_fg_TiO_2_; formerly registered as a food additive within the European Union (EU) as E171) as a relevant material for oral ingestion and contact with the intestinal epithelium. _fg_TiO_2_ is not intentionally manufactured as ENM as per the European Commission’s recommended definition^26^ but contains a fraction of nanosized particles that account for 20 to >70% of the material.^27^ TiO_2_ is no longer permitted as a food additive in the EU due to concerns regarding its DNA-damaging and pro-inflammatory potential,^28,29^ while it remains in use in other countries, e.g., the USA, Canada, and Australia. In vivo, _fg_TiO_2_ exposure affected the intestinal microbial composition, induced intestinal inflammation, and enhanced the effects of bacterial infection.^29−32^ While Pinget et al.^30^ reported a reduction in mucins and SCFA in mice, Talbot et al.^33^ did not observe changes in SCFA content or mucin *O*-glycosylation in rats. _fg_TiO_2_ is readily absorbed and was reported to cause adverse effects in intestinal in vitro cultures, including oxidative stress, DNA damage, barrier disruption, changes in mucus secretion and mucin expression, and cytokine release.^34−36^ However, neither the influence of butyrate nor ongoing inflammatory conditions on _fg_TiO_2_-induced effects has been investigated to date. We have tested _fg_TiO_2_ in Caco-2 cells, a colon adenocarcinoma cell line as a model for the intestinal epithelium, in a pristine state and following a pretreatment mimicking the varying pH conditions of the gastro-intestinal passage. The pretreated _fg_TiO_2_ was further investigated in a complex triple-culture model combining Caco-2, HT29-MTX-E12, and differentiated THP-1 cells as model cell lines for enterocytes, goblet cells, and macrophages. The advanced model was applied in a stable state representing the healthy intestine as well as after induction of inflammation to mimic a damaged intestine.

## 2 Experimental Procedures

### 2.1 Reagents and Materials

Sigma-Aldrich/Merck: d-glucose, trypsin, fetal bovine serum (FBS, F7524), nonessential amino acids, lipopolysaccharides (LPS, L4391), phosphate buffered saline (PBS), phorbol-12-myristate-13-acetate (PMA), bovine serum albumin (BSA), interferon gamma (IFNγ, SRP3058), and sodium butyrate (B5887).

Thermo Fisher Scientific: minimum essential medium (MEM + NEAA; 10370-021), Dulbecco’s modified Eagle medium (DMEM, high glucose, l-glutamine; 41965-039), Roswell Park Memorial Institute (RPMI)1640 (RPMI, l-glutamine, 25 mM HEPES; 52400-041), l-glutamine, sodium pyruvate, 2-mercaptoethanol (2-ME, 50 mM), mucin (MUC)5AC antibody (MA5-12178), Zonula Occludens (ZO)-1 antibody (617300), Hoechst 33342 (H3570), AlexaFluor 488 (A28175), AlexaFluor 594 (A11037), and ProLong Gold Antifade mounting medium (P36934).

_fg_TiO_2_: E171 (HOMBITAN FG; purity 99.5%, Venator, Germany) was kindly provided by the Korean Research Institute of Standards and Science (KRISS) and was previously characterized extensively.^37,38^ The primary particle size was determined to be ∼150 nm by TEM. The hydrodynamic diameter in water roughly doubled to 301 nm on average. As demonstrated by Di Cristo et al.,^39^ the material was minimally affected by pretreatment mimicking the gastrointestinal passage. Neither the morphology nor the primary particle size or crystallinity of the material was affected. A low pH environment mimicking the gastric passage caused the material to aggregate/agglomerate, which persisted after increasing the pH in the solution simulating the intestinal environment (see Figure S1 and Han et al.^37^). In accordance with the _fg_TiO_2_ isoelectric point,^33^ the particles carried a slightly positive surface charge in strongly acidic artificial gastric fluids and overall were negatively charged in pH neutral cell culture medium.

Selected experiments were also conducted using zinc oxide (ZnO; NM110, JRC repository, Ispra, Italy) and polyvinylpyrrolidone (PVP)-coated silver ENM (Ag-PVP ENM; Sigma, catalog number 576832). The materials were characterized by Thongkam et al.^40^ and Kämpfer et al.,^41^ respectively.

### 2.2 Electron Paramagnetic Resonance Spectroscopy

Electron paramagnetic resonance (EPR) spectroscopy was used to measure the capacity of _fg_TiO_2_ to generate hydroxyl radicals (^•^OH) in the presence of hydrogen peroxide.^42^ The samples were either suspended in ultrapure H_2_O and sonicated for 10 min using a Branson Sonifier 450 at a duty cycle of 0.2 s and an output of 240 W (pristine TiO_2_) or suspended in fluids mimicking the gastric and intestinal pH conditions as described in Section 2.4 (pretreated _fg_TiO_2_). Of the suspension, 25 μL was mixed with hydrogen peroxide (125 mM, 25 μL) (Sigma-Aldrich/Merck) and the spin trap 5,5-dimethyl-1-pyrroline-*N*-oxide (DMPO) (25 mM, 50 μL) (Cayman Chemical). Ultrapure water mixed with H_2_O_2_ and DMPO served as a negative control. Fly ash (MAT41/EVA, IUF; van Maanen et al.^43^) and ZnO ENM were included as positive controls. The mixture was shaken at 37 °C and protected from light for 15 min. Of the mix, 50 μL was drawn into a glass capillary and transferred to a Miniscope MS 200 EPR spectrometer (Magnettech, Germany). The following measurement settings were applied: Sweep: 100 G, sweep time: 30 s, number of scans: 3. Two replicates were prepared for each sample. For quantification, the mean amplitude of the DMPO–OH quartet was calculated and expressed as a fold change against the negative control.

### 2.3 Cell Culture

Cell lines: The complete culture media for the cell lines Caco-2 (DSMZ, ACC169), HT29-MTX-E12 (hereinafter “E12”; ECACC, 12040401), and THP-1 (ATCC, TIB-202) are summarized in Table 1. All cells were cultured for a minimum of three passages after thawing before experimental use. Caco-2 and E12 cells were used for up to 25 passages and THP-1 cells for 15 passages.

**Table 1:** Culture Medium Composition

cell line	culture medium and substitutes
Caco-2	MEM with nonessential amino acids, 20% heat-inactivated FBS (or 1% for serum-reduced medium), 1% penicillin/streptomycin, 0.4 mM l-glutamine
HT29-MTX-E12	DMEM with high glucose and l-glutamine, 10% heat-inactivated FBS, 1% penicillin/streptomycin, 1% nonessential amino acids
THP-1	RPMI 1640 with l-glutamine and 25 mM HEPES, 0.7% d-glucose, 1 mM sodium pyruvate, 1% penicillin/streptomycin, 10% heat-inactivated FBS, 50 nM 2-mercaptoethanol (2 ME)

Caco-2 monocultures: Caco-2 cells were seeded at a concentration of 1 × 10^4^ cells cm^–2^ in 100 μL in 96-well plates (Falcon) or 6 × 10^4^ cells cm^–2^ in 1 mL in 24-well plates (Falcon) and incubated for 24 h. The medium was exchanged for a serum-reduced culture medium containing 1% FBS and incubated for 24 h before exposure experiments were conducted.

Triple cultures: Triple cultures were established on transwell inserts (12-well, PET, 1 μm pore size; Falcon, 353103) using Caco-2, E12, and THP-1 cells, as described previously.^41^ Epithelial cocultures of Caco-2 and E12 cells were seeded at a 9:1 ratio and maintained for 21 days. The basolateral medium was gradually transitioned from the Caco-2 MEM-based to the THP-1 RPMI-based culture medium. Stable triple cultures representing the healthy human intestine were established by adding 1.8 × 10^5^ PMA-differentiated (100 nM, 24 h) THP-1 cells to the basolateral compartment; for the inflamed model, the epithelial transwell cultures were primed with IFNγ (10 ng mL^–1^) for 24 h. The PMA-differentiated THP-1 cells were preactivated with LPS and IFNγ (10 ng mL^–1^ each) before the primed epithelial coculture transwells were placed onto the well. The triple cultures were maintained for 48 h.

### 2.4 Exposures

Preparations: For experiments on pristine _fg_TiO_2_, the material was suspended in ultrapure water at a concentration of 4 mg mL^–1^ and sonicated using a cup horn sonifier (Sonifier 450, Branson Ultrasonics, USA) at a duty cycle of 0.2 s and output of 240 W for 10 min. For the pretreatment simulating the pH conditions of the gastrointestinal passage, _fg_TiO_2_ (hereinafter “pre-treated”) was dispersed as described previously.^44^ Briefly, the material was weighed at 5–10 mg and suspended in artificial gastric solution (34 mM NaCl/HCl, pH 2.7) to a concentration of 4 mg mL^–1^ and incubated for 30 min at 37 °C. Subsequently, the suspension was neutralized by adding 0.12 times the volume of artificial intestinal solution (1.68 g of NaCO_3_, 7.16 g of NaHCO_3_, and 4 g of NaCl in 2 L of H_2_O). The suspension was incubated again for 30 min at 37 °C, resulting in a starting concentration of 3.57 mg mL^–1^_fg_TiO_2_. The exposure concentrations were chosen (1) for comparability to existing studies using the same or similar concentrations^25,41,45^ and (2) as they represent realistic average exposure concentrations of adults according to the European Food Safety Authorities safety assessment of titanium dioxide.^28^ For more information on how the exposure concentrations were derived, please refer to Supporting Information (Section 1, p. 2).

For butyrate exposure, a stock concentration of 100 mM was prepared in ultrapure H_2_O. A nontoxic concentration of butyrate was established for the coincubation experiments. In proliferating Caco-2 monocultures, concentrations equal to or above 2 mM significantly reduced the metabolic activity after 24 h (Figure S2). Therefore, the following experiments in Caco-2 monocultures were conducted using a concentration of 1 mM butyrate. In each experiment, a positive and negative control was included. The negative control was composed of a serum-reduced culture medium containing the maximum volume of dispersant, i.e., ultrapure water or a mixture of the digestion simulants. The positive control was chosen depending on the end point and the assay.

**Scheme 1:**
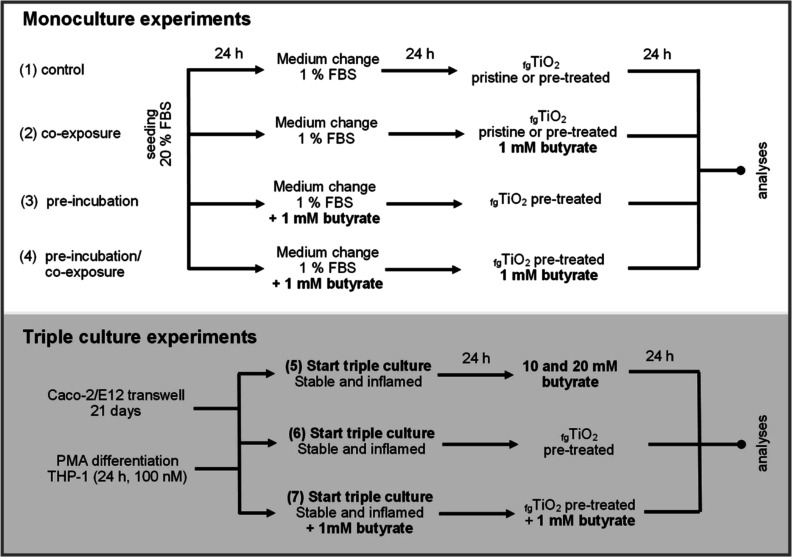
Experimental Setup and Exposure Conditions Experiments were conducted in Caco-2 monocultures (1–4) and triple cultures of Caco-2, E12, and PMA-differentiated THP-1 cells in stable and inflamed-like states (5–7). As control (1), Caco-2 cells were grown for 48 h before exposure to pristine and pre-treated _fg_TiO_2_ for 24 h. The results were compared to monocultures exposed to _fg_TiO_2_ in the presence of 1 mM butyrate (2), pre-incubated for 24 h with 1 mM butyrate before exposure to _fg_TiO_2_ (3), or pre-incubated and subsequently co-exposed to _fg_TiO_2_ and 1 mM butyrate for 24 h (4). Before _fg_TiO_2_ exposures, the triple cultures response to higher butyrate concentrations (10 and 20 mM) was tested (5). For exposure experiments, the cultures were established and left to equilibrate for 24 h before pre-treated _fg_TiO_2_ was added apically (6). To investigate the effect of butyrate, the triple cultures were established and pre-incubated with 1 mM butyrate from the apical side for 24 h and subsequently co-exposed to pre-treated _fg_TiO_2_ and 1 mM butyrate for 24 h (7).

Exposure conditions: In Caco-2 monocultures, four different exposure setups were tested, which are summarized in Scheme 1 (1–4). Cells were exposed to pristine or pretreated _fg_TiO_2_ (0–80 μg cm^–2^) for 24 h (1). After establishing a nontoxic incubation concentration for butyrate (i.e., 1 mM), the effects of butyrate coexposure ((2), 24 h with _fg_TiO_2_), preincubation ((3), 24 h before exposure to _fg_TiO_2_), or preincubation and coexposure (4) were tested. Since differentiated cells are more robust than proliferating cells,^41^ the adequacy of the butyrate concentration was tested in the advanced triple cultures (5) before conducting _fg_TiO_2_ exposure experiments (6–7). We initially tested higher butyrate concentrations (i.e., 10 and 20 mM) that more closely resemble the physiological concentrations in the intestine.^46^ However, both concentrations disrupted the barrier integrity of the stable and inflamed model (Figure S3A,B), induced lactate dehydrogenase (LDH) release (Figure S3C), and a dose-dependent increase in gene expression for MUC2, MUC13, and IL8 (Figure S3D,E), while no changes were detected in the release of IL1β, IL8, and tumor necrosis factor alpha (TNFα) (Figure S4). Therefore, butyrate triple culture experiments were also conducted with a concentration of 1 mM. Triple cultures were established and equilibrated for 24 h, before being apically exposed to 10 or 80 μg cm^–2^ pretreated _fg_TiO_2_. To test the influence of butyrate, the triple cultures were established with 1 mM butyrate on the apical side and equilibrated for 24 h, before coexposure to 10 or 80 μg cm^–2^_fg_TiO_2_ and 1 mM butyrate was started. Untreated triple cultures were used as “stable control” and “inflamed control”.

### 2.5 Cytotoxicity

Metabolic activity: in Caco-2 monocultures seeded on 96-well plates, the metabolic activity was quantified by a WST-1 assay. The cells were seeded and treated as described in Sections 2.3 and 2.4, respectively, and incubated for 24 h. Triton X-100 (0.5%) served as a positive control. Subsequently, 10 μL of WST-1 solution was added to each well and incubated for 1 h at 37 °C. The absorption was measured spectrophotometrically (Multiskan Go) at 450 and 630 nm. The results were corrected for the cell-free background and the positive control background and expressed in relation to the negative control.

LDH assay: in triple cultures, cytotoxicity was detected by quantification of LDH. Apical and basolateral supernatants (50 μL) were transferred onto a 96-well plate, combined with 150 μL of a reaction mix containing 5 mL of TRIS buffer (Tris–HCl, Trisbase, 200 mM, pH 8, Sigma), 5 mL of lithium l-lactate (98 mg, Sigma), 200 μL of iodonitrotetrazolium (INT, 6.6 mg, Sigma), 200 μL of phenazine methosulfate (PMS, 1.8 mg, Sigma), and 5 mL of β-nicotinamide adenine dinucleotide sodium salt (NAD, 17.2 mg, Sigma) and incubated for 5 min at 37 °C, 5% CO_2_. The reaction was stopped with 50 μL of 1 M H_2_SO_4_ (Sigma) and the absorbance read spectrophotometrically at 490 and 680 nm. The results were background corrected and expressed as optical density values. As particulate materials may interfere with the assay, the quantification of LDH from lysed cells was investigated for 10 and 80 μg cm^–2^_fg_TiO_2_ and incubation periods of ∼1 min and 4 and 24 h. No differences from the negative control were detected for any of the time points (data not shown).

Epithelial barrier integrity: Barrier integrity was monitored throughout differentiation as well as after the establishment of triple cultures and exposures to butyrate and _fg_TiO_2_ by measuring the transepithelial electrical resistance (TEER) using a voltohmmeter (EVOM, World Precision Instruments) and a chopstick electrode (STX2, World Precision Instruments). The transwell plate was allowed to temperature equilibrate for ∼2 min before measurement. Cell-free transwell inserts were included as blanks. The measured resistance was corrected for the blank values and surface area of transwell inserts (0.9 cm^2^) and expressed in relation to the unexposed stable triple culture control.

### 2.6 Quantification of Cytokine Release

Cytokines were quantified by enzyme-linked immuno-sorbent assay using DuoSet antibody kits (RnD) as previously described.^41^ IL8 was quantified in undiluted supernatants from Caco-2 monocultures after 24 h exposure to pristine and pretreated _fg_TiO_2_ in the presence or absence of 1 mM butyrate. In triple culture, IL8, IL1β, and TNFα were quantified in the basolateral supernatants after 48 h triple culture and 24 h exposure to pretreated _fg_TiO_2_ in the presence or absence of 1 mM butyrate. For IL8, the basolateral supernatants were diluted 1:10 in 1% BSA/PBS. The primary antibody was incubated on high-protein-binding 96-well plates (Nunc) in coating buffer (0.1 M NaHCO_3_, pH 8.2) at room temperature (RT) overnight. After blocking with 3% BSA/PBS, the samples were incubated for 2 h at RT. The secondary antibody was incubated in 1% BSA/PBS for 2 h at RT. Following incubation with horseradish peroxidase (1:40 in 1% BSA/PBS for 30 min, RT), 100 μL of TMB Peroxidase EIA Substrate Kit (BioRad) was added and incubated for 10–20 min at RT before the reaction was stopped using 50 μL of 1 M H_2_SO_4_. The absorbance was measured spectrophotometrically at 450 nm and the standard curve plotted as a four-parameter logistic fit.

### 2.7 Gene Expression Analysis

Gene expressions for the mucins MUC1, MUC2, MUC5AC, MUC13, and MUC20, IL8, DNA damage repair proteins oxoguanine glycosylase 1 (OGG1), X-ray repair cross-complementing protein 1 (XRCC1) and apurinic/apyrimidinic endonuclease 1 (APE1) as well as the oxidative stress markers heme oxygenase 1 (HMOX1) and γ-glutamylcysteine synthetase (γGCS) were assessed in the epithelial cells of the triple culture by quantitative real-time PCR, as described before.^41^ Briefly, the RNA concentration was determined by measuring the optical densities at 260 and 280 nm. The samples were treated with amplification grade DNase I. Two replicates of 0.5 μg of RNA were reverse transcribed using the iScriptTM cDNA synthesis kit. A no reverse transcriptase control (nRTc) with one replicate of 0.5 μg of RNA was performed in parallel as a control for residual DNA. The duplicate cDNA samples were pooled before cDNA and nRTc were diluted in nuclease-free water by a factor of 15. The iQTM SYBR Green Supermix was used for qPCR reactions. The primer sequences summarized in Table 2 were used and normalized against β-actin. qPCR reactions were performed, and melt curves were generated in triplicate for cDNAs and one replicate for nRTcs using a MyiQTM Single-Color Real-Time PCR Detection System (Bio-Rad, Hercules, USA). CT values were determined using Bio-Rad iQ5 software (v2.1). Changes in the gene expression were calculated by using the ΔΔCT method.

**Table 2:** Sequences, Product Lengths, and Concentrations of the Used Primers

gene	sequence (5′ to 3′)	amplicon (bp)	concentration (nM)
β-actin	FW CCTGGCACCCAGCACAAT	70	60
	RV GCCGATCCACACGGAGTACT		60
MUC1	FW AGACGTCAGCGTGAGTGATG	139	37.5
	RV GACAGCCAAGGCAATGAGAT		37.5
MUC2	FW GTCCGTCTCCAACATCACCT	287	60
	RV GCTGGCTGGTTTTCTCCTCT		60
MUC5AC	FW CAGCACAACCCCTGTTTCAAA	100	60
	RV GCGCACAGAGGATGACAGT		37.5
MUC13	FW CAGAGACAGCCAGATGCAAA	175	60
	RV CGGAGGCCAGATCTTTACTG		37.5
MUC20	FW GTGCAGGTGAAAATGGAGGT	152	60
	RV ACGCAGTAAGGAGACCTGGA		37.5
IL8	FW ACTCCAAACCTTTCCACCC	168	60
	RV CCCTCTTCAAAAACTTCTCCAC		60
OGG1	FW ACATTGCCCAACGTGACTACA	145	200
	RV GCACTGAACAGCACCGCTT		200
XRCC1	FW AGAATGGGGAAGACCCGTAT	178	200
	RV GCTGTGACGTATCGGATGAG		200
APE1	FW CTGCCTGGACTCTCTCATCAATAC	118	200
	RV CCTCATCGCCTATGCCGTAAG		200
HMOX1	FW ATGACACCAAGGACCAGAGCC	151	200
	RV GTAAGGACCCATCGGAGAAGC		200
γGCS	FW TTGCAGGAAGGCATTGATCA	101	200
	RV GCATCATCCAGGTGTATTTTCTCTT		200

### 2.8 Immunocytochemical Staining

After 48 h of triple culture, the epithelial transwell cultures were fixed in 4% paraformaldehyde (PFA) from the apical and basolateral sides for 20 min at RT. The fixed layer was stained for MUC5AC, the most prominently expressed mucin in E12 cells, the tight-junction protein ZO-1, and nuclei. The cells were permeabilized with 0.1% Triton X-100 in PBS for 5 min at RT and blocked against unspecific binding with 3% BSA in PBS. Filters were incubated with MUC5AC primary (2 μg mL^–1^) and ZO-1 antibodies in 1% BSA/PBS for 1 h at RT. Subsequently, cells were counterstained with AlexaFluor 488 antibody (1:300), AlexaFluor 594 antibody (1:300), and Hoechst 33342 (0.5 μg mL^–1^) for 30 min at 37 °C. Filters were placed on microscopy slides, mounted with Prolong Gold Antifade, and sealed with a coverslip. Microscopic evaluation was performed using a Zeiss Axio Imager.M2 at 10× magnification.

### 2.9 Statistical Analysis

Data analysis was performed with Microsoft Excel, while the results were illustrated and statistically analyzed with GraphPad Prism (Version 9). Experiments were performed in three independent runs with 3–4 biological replicates in Caco-2 monocultures and 2 biological replicates in triple cultures, unless stated otherwise. Statistical analysis was performed as one-way analysis of variance (ANOVA) with Dunnett’s post hoc test or unpaired *t*-test, as specified in the figure legends. A *p*-value of <0.05 was considered statistically significant.

## 3 Results

### 3.1 Oxidative Potential of _fg_TiO_2_ in Cell-Free Environment

As several studies previously reported _fg_TiO_2_ to exert adverse effects through oxidative stress,^34,47^ we investigated the material’s oxidative potential in a cell-free environment both in pristine and pretreated states. While both particulate controls, fly ash and ZnO ENM, demonstrated a significant capacity for ^•^OH formation, no effect was observed for _fg_TiO_2_ (Figure S5).

### 3.2 Co-exposure of Caco-2 Monocultures to _fg_TiO_2_ and Butyrate

**Figure 1:**
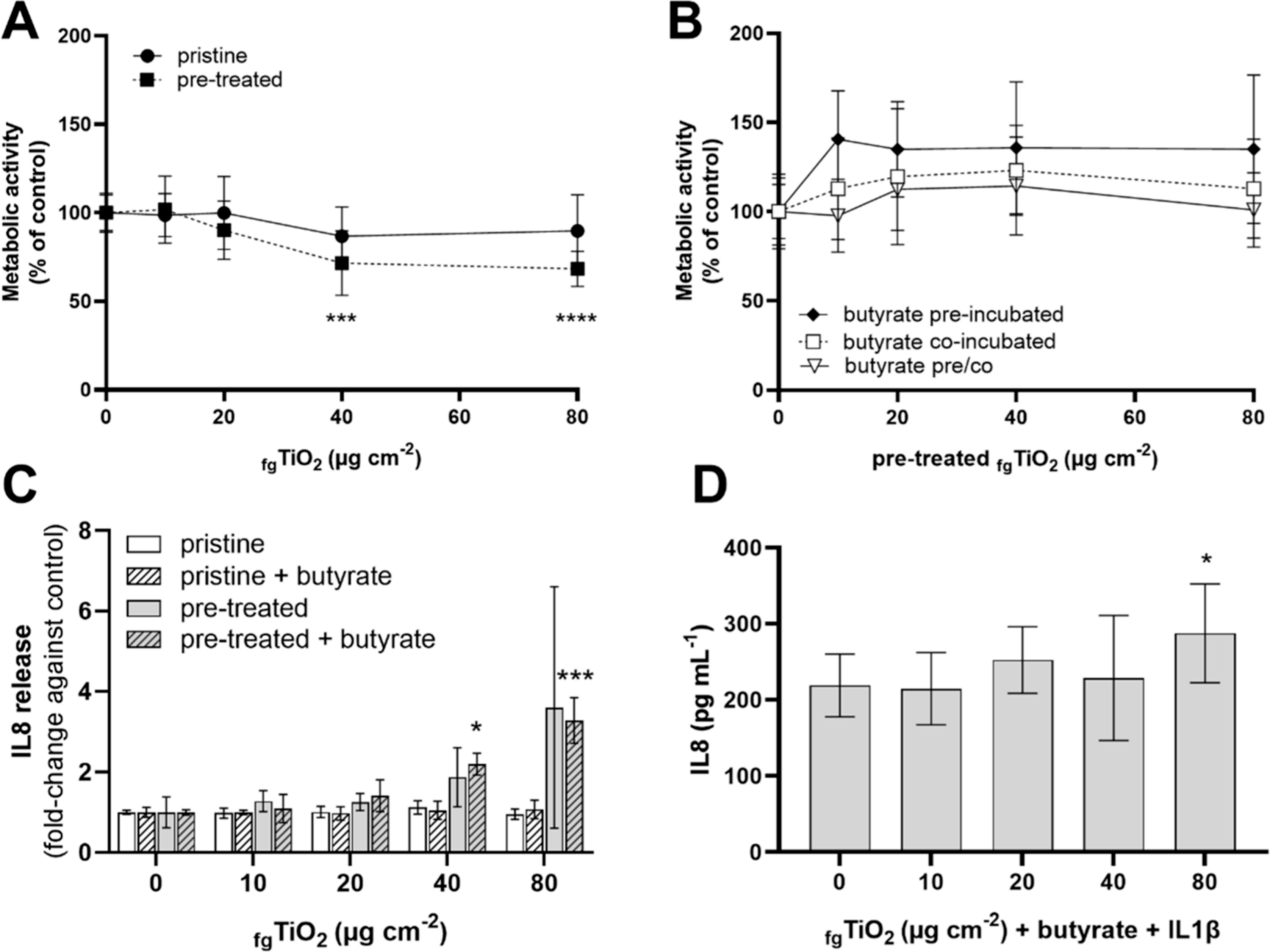
Effect of butyrate on the cytotoxicity and IL8 release induced by _fg_TiO_2_. (A) Metabolic activity of cells was measured after 24 h exposure to 0–80 μg cm^–2^ pristine or pretreated _fg_TiO_2_ and (B) pretreated _fg_TiO_2_ combined with different butyrate treatments. For butyrate treatments, cells were preincubated with butyrate before _fg_TiO_2_ exposure, coexposed with _fg_TiO_2_ and butyrate, or preincubated and coexposed. IL8 release was measured after 24 h exposure to (C) pristine or pretreated _fg_TiO_2_ in the absence or presence of 1 mM butyrate or (D) after 24 h exposure to pretreated _fg_TiO_2_ and butyrate in cells activated with 10 ng mL^–1^ IL1β. (Mean ± SD, *N* = 3, *p ≤ 0.05/***p ≤ 0.005/****p ≤ 0.001 compared to corresponding control by one-way ANOVA and Dunnett’s post hoc test.).

Pristine and pretreated _fg_TiO_2_ were first tested in proliferating Caco-2 monocultures. While no effect on cell viability was observed after 24 h exposure to pristine _fg_TiO_2_, pretreated _fg_TiO_2_ significantly reduced the metabolic activity at exposure concentrations of 40 and 80 μg cm^–2^ to 71 and 68%, respectively (Figure 1A). To elucidate, if the effect could be altered by butyrate, the cells were treated as described in Scheme 1 ((2)-(4)) and exposed to pretreated _fg_TiO_2_ for 24 h. The previously observed _fg_TiO_2_-induced reduction in metabolic activity was inhibited under all three butyrate treatment protocols (Figure 1B). This protective effect of butyrate was not exclusive to _fg_TiO_2_ but also observed in Ag-PVP- but not ZnO-exposed Caco-2 monocultures (Figure S6A–D and Table S1).

Subsequently, the IL8 release was investigated for pristine and pretreated _fg_TiO_2_ alone and coexposed with butyrate. As it was previously reported that butyrate can inhibit the Caco-2 cytokine response^22,48^ we first investigated if the three different butyrate treatments (Scheme 1 (2–4)) affect the IL1β-induced IL8 release (Figure S7). IL1β stimulation led to a significant release of IL8 (330 ± 88 pg mL^–1^), and all three tested butyrate treatments significantly reduced IL8 release in IL1β-activated Caco-2 cells by 39 to 51%. In the absence of IL1β, none of the butyrate treatments caused a significant change in IL8 release compared to the control (24 ± 3 pg mL^–1^). While pristine _fg_TiO_2_ did not induce IL8, a dose-dependent increase in IL8 release was quantified after exposure to pretreated _fg_TiO_2_, which reached statistical significance only in the presence of butyrate (Figure 1C). As we have previously observed that butyrate inhibits the IL8 release in IL1β-activated Caco-2 cells, we tested if this effect prevails in the presence of a second stimulus. Therefore, Caco-2 cells were activated with IL1β and coexposed to pretreated _fg_TiO_2_ and butyrate (Figure 1D). After 24 h, a slight but significant increase in IL8 release was detected at an exposure concentration of 80 μg cm^–2^.

### 3.3 Butyrate-Treated Intestinal Triple Cultures

Due to the absence of effects by pristine _fg_TiO_2_, the subsequent intestinal triple culture experiments were only conducted with pretreated _fg_TiO_2_.

As described in Scheme 1 (7), the triple cultures were established with 1 mM butyrate in the apical medium. After 24 h, the apical supernatants were exchanged for a serum-reduced medium containing 1 mM butyrate. When butyrate-treated triple cultures were compared to control triple cultures, no effects were seen on barrier integrity (Figure S8A), mucin expression in epithelial cells of the inflamed compared to the stable model (Figure S8B), and LDH release (data not shown). Cytokines in basolateral supernatants were highly comparable between butyrate-incubated and control cultures (Figure S9A), while the apical release of IL8 was increased in the inflamed model in the presence of butyrate (Figure S9B). In line with the strong reduction in barrier integrity, the ZO-1 network of the epithelial barrier was strongly disrupted in the inflamed model, regardless of the presence of butyrate (Figure S10).

### 3.4 Effects of _fg_TiO_2_ and _fg_TiO_2_/Butyrate in Intestinal Triple Cultures

#### 3.4.1 Barrier Integrity and LDH Release

**Figure 2:**
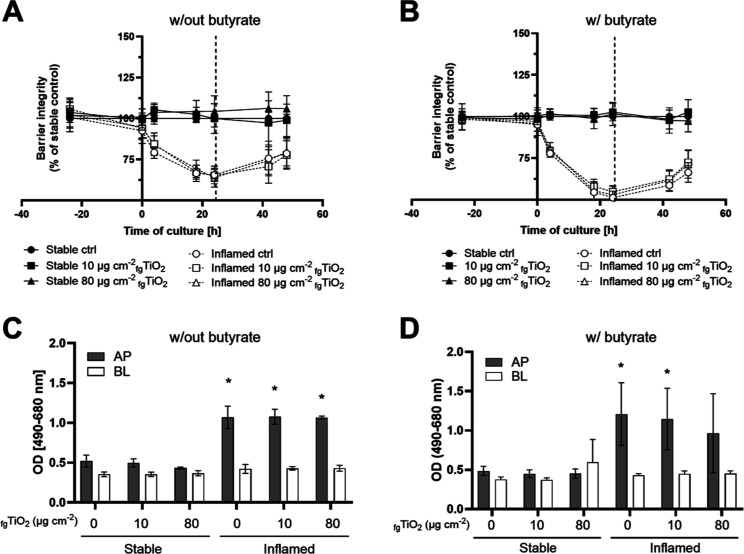
Effect of _fg_TiO_2_ on barrier integrity (A,B) and LDH release (C,D) in stable and inflamed triple cultures in the absence or presence of butyrate. Triple cultures were exposed to 10 or 80 μg cm^–2^ pretreated _fg_TiO_2_ for 24 h (A,C) or following preincubation and in the presence (B,D) of 1 mM butyrate. (mean ± SD, *N* = 3; dotted line in graphs A and B marks start of _fg_TiO_2_ exposure; AP: apical supernatants, BL: basolateral supernatants; ctrl: unexposed controls of the stable and inflamed triple cultures; w/out = triple cultures established and exposed without butyrate, w/ = triple cultures established and exposed with butyrate; barrier integrity of inflamed cultures was significantly reduced compared to the stable model from measurement point t_4_ and thereafter; *p ≤ 0.05 compared to corresponding stable triple culture control by one-way ANOVA and Dunnett’s post hoc test.).

Following the basic characterization, coexposures of butyrate and pretreated _fg_TiO_2_ were conducted and compared to triple cultures exposed to pretreated _fg_TiO_2_ alone. Four hours after initiation of the triple culture, the TEER was significantly reduced in inflamed triple cultures both in the absence (*p* ≤ 0.001) and presence (*p* ≤ 0.001) of butyrate (Figure 2, A,B, significance not indicated in graph). While the TEER started to recover again after 24 h, it remained below the stable control at the end of the triple culture (*p* = 0.003 and *p* ≤ 0.001 for cultures without and with butyrate, respectively). Neither the exposure to pretreated _fg_TiO_2_ alone (A) nor the coincubation with butyrate and pretreated _fg_TiO_2_ (B) induced observable effects in barrier integrity in either model.

In accordance with the reduction in barrier integrity, the apical LDH release was increased in inflamed triple cultures (Figure 2, C&D). While LDH activity was significantly increased after exposure to 10 and 80 μg cm^–2^ pretreated _fg_TiO_2_ (C), it failed to reach statistical significance in inflamed triple cultures coexposed to 80 μg cm^–2^_fg_TiO_2_ and butyrate (D). No change in LDH was observed in the supernatants of the stable model and the basolateral supernatants of the inflamed model. The increase in apical LDH release is commonly observed in the inflamed triple culture and also serves as a marker for reproducibility of the model. It indicates epithelial cell necrosis in response to the high release of pro-inflammatory cytokines by LPS/IFNγ-activated THP-1 cells in the basolateral compartment.^41^

#### 3.4.2 Cytokine Release

**Figure 3:**
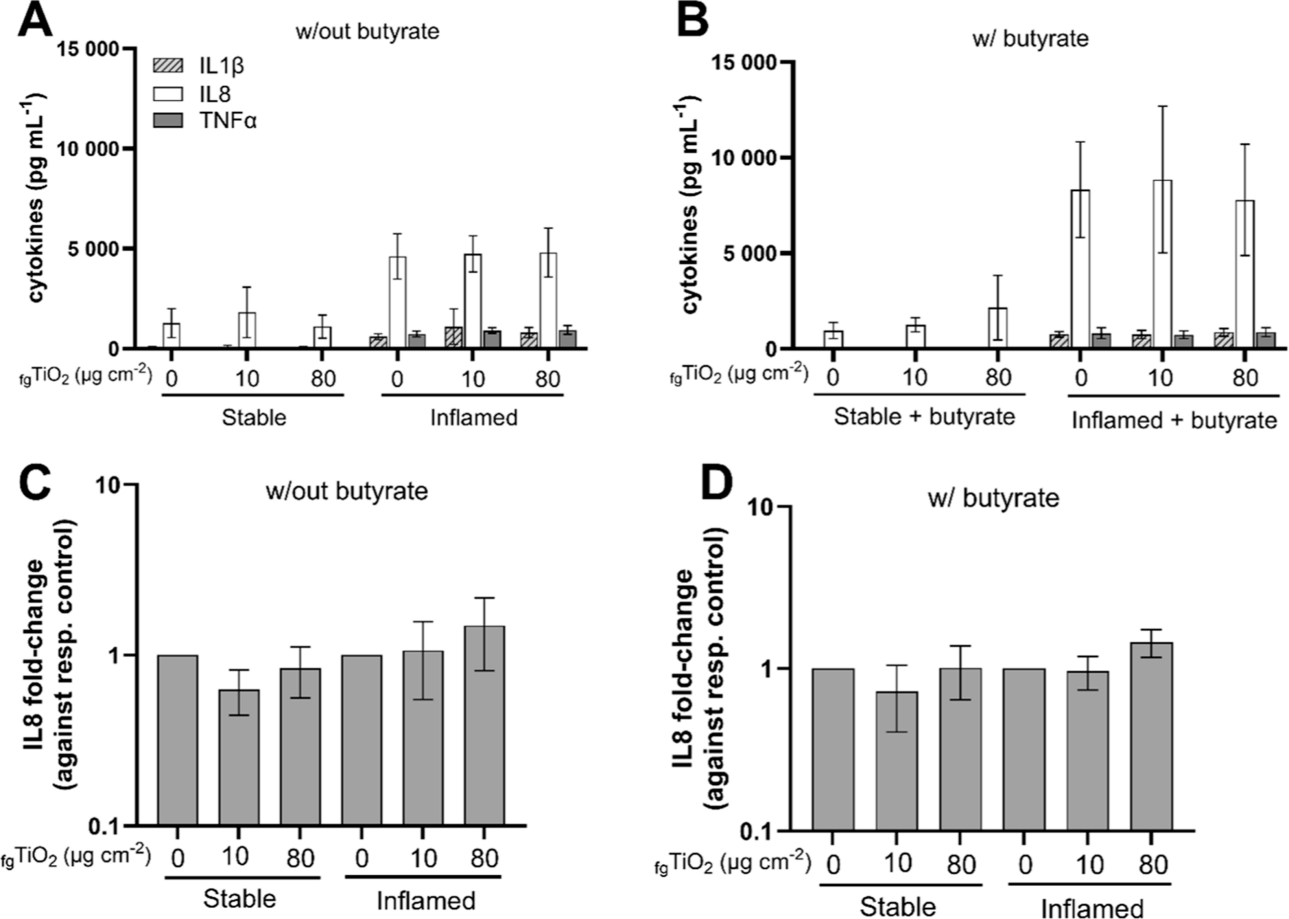
Cytokines in stable and inflamed triple cultures after 24 h exposure to pretreated _fg_TiO_2_ alone (A,C) or with preincubation and coexposure to 1 mM butyrate (B,D). (A,B) The release of IL8, IL1β, and TNFα was quantified in basolateral supernatants. (C,D) The gene expression of IL8 was quantified in epithelial cells (Caco-2 and E12) of stable and inflamed triple cultures and expressed against the respective control. (mean ± SD, A,C: *N* = 4, B,D: *N* = 3; w/out = triple cultures established and exposed without butyrate, w/ = triple cultures established and exposed with butyrate).

The release of IL1β, IL8, and TNFα was quantified in apical and basolateral supernatants (Figure 3, A&B and Table 3). In the stable model, only IL8 was detectable, while the concentrations of all three cytokines were significantly increased in the basolateral supernatants of inflamed triple cultures (not indicated in graphs). Neither _fg_TiO_2_ nor butyrate-_fg_TiO_2_ coexposure induced significant changes. In the apical supernatants, only IL8 was detected (Table 3). In stable triple cultures, no change was observed. In butyrate-treated inflamed cultures, the apical IL8 release was increased compared to the inflamed control but with a large standard deviation. Therefore, the IL8 gene expression was also analyzed in the epithelial cells of both models (Figure 3C,D). No statistically significant changes were detected.

**Table 3:** IL8 in Apical Supernatants^a^

	stable		inflamed	
pretreated TiO_2_ (μg cm^–2^)	w/out butyrate	w/butyrate	w/out butyrate	w/butyrate
0	31.7 ± 10.3	33.4 ± 14.9	247.0 ± 22.01	517.7 ± 246.5
10	41.67 ± 16.5	33.1 ± 14.8	302.4 ± 64.9	388.13 ± 149.3
80	29.4 ± 12.8	37.5 ± 21.1	299.3 ± 67.1	465.3 ± 221.3

a Mean ± SD, *N* = 3, in pg mL^–1^.

#### 3.4.3 Mucin Expression and Mucus Secretion

**Figure 4:**
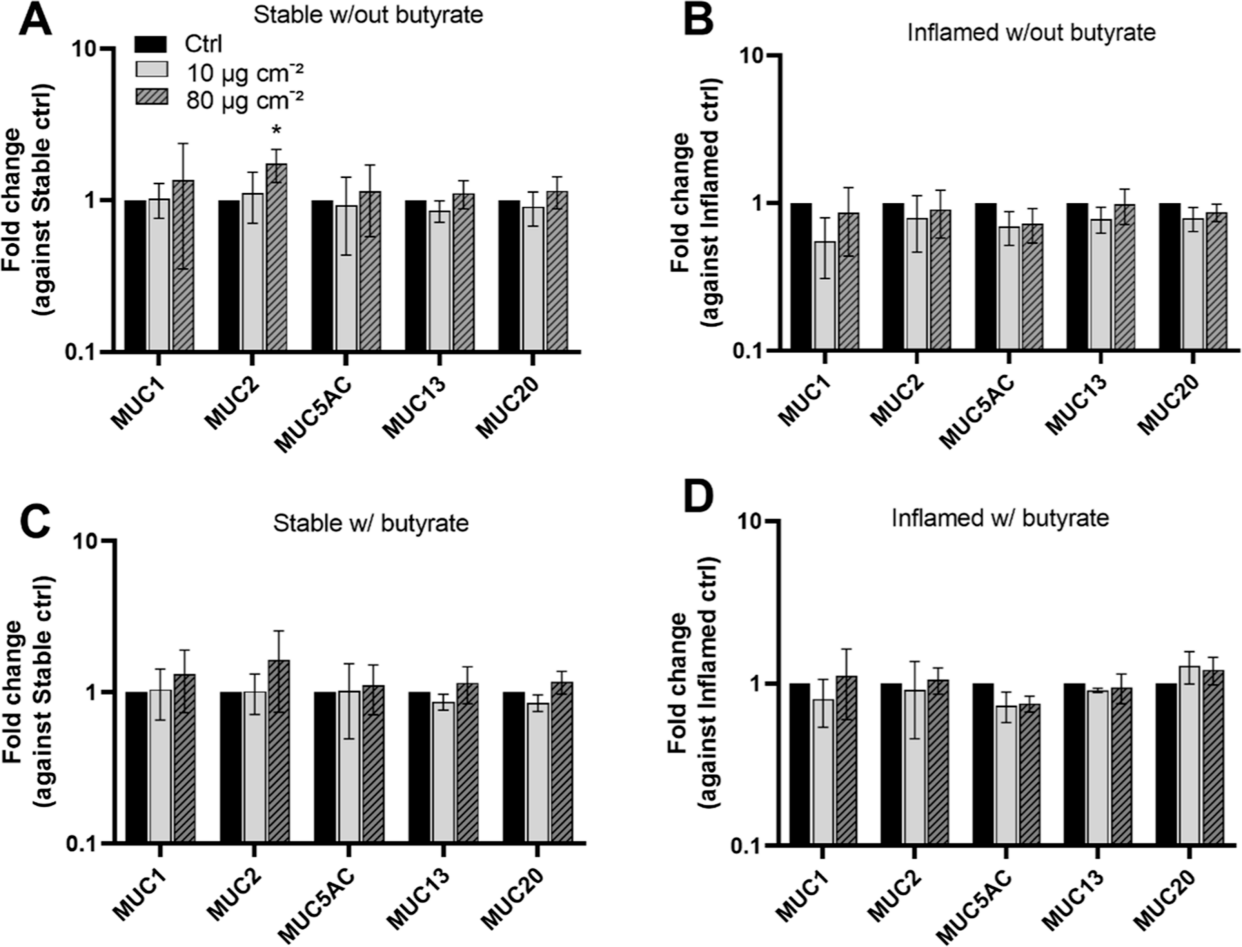
Gene expression of MUC1, MUC2, MUC5AC, MUC13, and MUC20 in epithelial cells of stable and inflamed triple cultures after 24 h exposure to pretreated _fg_TiO_2_ alone (A,B) or with preincubation and coexposure to 1 mM butyrate (C,D). The results were normalized to the corresponding unexposed control and β-Actin as the reference gene. The depicted fold changes were derived from the ΔΔCT-values. (mean ± SD, A,B: *N* = 4; C,D: *N* = 3; ctrl: unexposed controls of the stable and inflamed triple cultures; w/out = triple cultures established and exposed without butyrate, w/ = triple cultures established and exposed with butyrate; *p ≤ 0.05 compared to corresponding control by *t*-test).

The expression of five mucins, MUC1, MUC2, MUC5AC, MUC13, and MUC20, was quantified in the epithelial cells of stable and inflamed triple cultures after exposure to _fg_TiO_2_ (Figure 4A,B) and coexposure to butyrate and _fg_TiO_2_ (C&D). While the expression of all mucins was significantly changed in the epithelial cells of the inflamed model compared to the stable model (Figure S8B), the exposure to _fg_TiO_2_ only induced minor effects. In the stable triple culture, 80 μg cm^–2^_fg_TiO_2_ induced an increase in MUC2 expression, which was statistically significant (*p* = 0.048) in the absence but not in the presence of butyrate.

**Figure 5:**
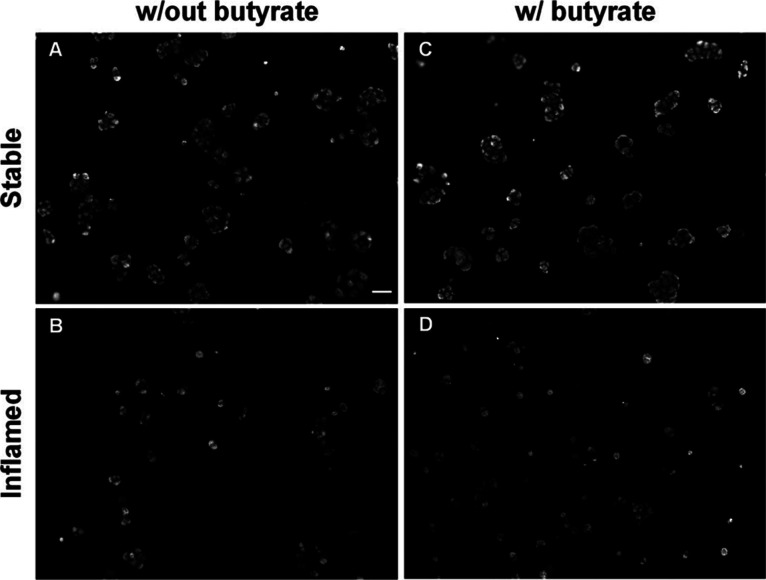
MUC5AC staining in epithelial transwell cultures of (A,B) stable and (C,D) inflamed triple cultures. The triple cultures were maintained for 48 h in the absence (A,C) or presence (B,D) of 1 mM butyrate. (10× magnification, scale bar = 50 μm; w/out = triple cultures established and exposed without butyrate, w/ = triple cultures established and exposed with butyrate).

MUC5AC is the most abundantly expressed mucin in E12 cells. While it is still prominently expressed in the epithelial cocultures of the stable model, it is strongly reduced in the inflamed model,^25^ which has been confirmed here (Figure S8B). The strong reduction was visualized by immunocytochemical staining for MUC5AC in the epithelial transwell cultures of the stable and inflamed models (Figure 5). In the stable model, a majority of larger clusters of MUC5AC-secreting cells is visible next to few isolated cells (A). In contrast, in the epithelial cocultures of the inflamed model (B), the overall quantity of MUC5AC was reduced and the cell clusters largely diminished. These results are in line with the previous analysis by Busch et al.^49^ The incubation with butyrate (C&D) did not affect the overall number of MUC5AC-expressing cells or the size of the cell clusters in the stable or inflamed model compared to the corresponding control.,.

#### 3.4.4 DNA Repair and Oxidative Stress

_fg_TiO_2_ has been critically discussed for its DNA-damaging potential.^28^ While other studies reported a direct quantification of DNA damage by alkaline comet assay,^47^ we could not generate robust data with the assay due to interferences of _fg_TiO_2_ that remained on the samples (Figure S11). Notably, we did not observe this problem with other TiO_2_ samples, e.g., P25, in previous studies.^41,50^ This limitation might be due to the intrinsic fluorescent properties of food-grade compared to P25 TiO_2_, as also described by Talbot et al.^33^

**Figure 6:**
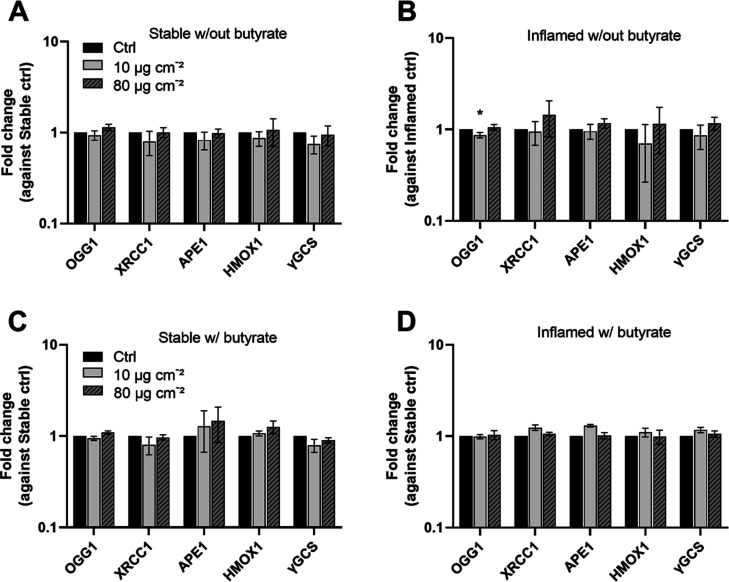
Gene expression of OGG1, XRCC1, APE1, HMOX1, and γGCS in epithelial cells of stable and inflamed triple cultures after 24 h exposure to pretreated _fg_TiO_2_ alone (A,B) or with preincubation and coexposure to 1 mM butyrate (C,D). The results were normalized to the corresponding unexposed controls and β-Actin as the reference gene. The depicted fold changes were derived from the ΔΔCT-values. (average ± SD, A,B: *N* = 4; C,D: *N* = 3; ctrl: unexposed controls of the stable and inflamed triple cultures; w/out = triple cultures established and exposed without butyrate, w/ = triple cultures established and exposed with butyrate; *p ≤ 0.05 compared to corresponding control by one-way ANOVA and Dunnett’s post hoc test.).

Therefore, we investigated the gene expression of the oxidative stress markers HMOX1 and γ-GCS as well as the DNA damage repair proteins, OGG1, XRCC1, and APE1 in the epithelial cells of stable and inflamed triple cultures (Figure 6). The relative mRNA expression of the investigated genes was significantly lower in the inflamed model compared to the stable model, irrespective of the absence and presence of butyrate (Figure S12). In both models, neither the exposure to _fg_TiO_2_ alone (A&B) nor in combination with butyrate (C&D) induced effects except for a minor but statistically significant reduction (*p* = 0.033) of OGG1 expression in inflamed triple cultures treated with 10 μg cm^–2^_fg_TiO_2_ (B).

## 4 Discussion

We showed that butyrate affects the IL8 response and inhibits cytotoxicity of pretreated _fg_TiO_2_ in proliferating monocultures of Caco-2 cells, while no effects of _fg_TiO_2_, butyrate, or a combination of both were observed in triple cultures in a healthy or inflamed state. As a byproduct of bacterial fermentation, butyrate is ubiquitously present at concentrations between 2 and 24 mmol kg^–1^ in the small and large intestines, respectively.^46,51^ Albeit its physiological importance for intestinal homeostasis, it has rarely been examined in *in vitro* hazard assessments, including particle-induced toxicity. The results presented suggest that the consideration of butyrate can significantly alter the outcomes depending on the test system used.

All three butyrate treatment protocols inhibited IL1β-induced IL8 release from Caco-2 monocultures. Since already the preincubation with butyrate was sufficient, we concluded that the effect is not related to an interaction, e.g., binding of IL1β, but linked to butyrate’s regulatory functions in the cells. Previous studies reported conflicting results. Weng et al.^52^ and Fusunyan et al.^53^ found 3–5 mM butyrate to enhance IL8 release in response to pathogen-associated molecular patterns, LPS, and IL1β in Caco-2 cells and nonmalignant enterocytes. In contrast, Böcker et al.^54^ demonstrated an inhibitory effect of 5 mM butyrate on IL1β-induced IL8 release in both Caco-2 and primary intestinal epithelial cells, while Li et al.^55^ reported an inhibitory effect on the release of IL6 but not IL8 in human endothelial cells. These contrasting results may be related to the different butyrate concentrations used as well as the cells’ differentiation stage. As Malago et al.^22^ showed, the cytoprotective and immunomodulatory effects of butyrate can change depending on the concentration used. While low concentrations (up to 1 mM) protected heat-shocked Caco-2 cells against Salmonella-induced IL8 release, no protective effect was observed at higher butyrate concentrations (>5 mM). Furthermore, both Fusunyan et al.^53^ and Weng et al.^52^ investigated more mature but not fully differentiated Caco-2 cells at 14- and 7 days postseeding, which have previously been shown to react differently to butyrate than undifferentiated Caco-2 cells^6,22^ as used by Böcker et al.^54^

Exposure to pretreated _fg_TiO_2_ significantly reduced the metabolic activity in Caco-2 monocultures, while this effect was not observed in butyrate-treated cells, suggesting a protective effect of butyrate. Butyrate was shown to enhance mitochondrial function and antioxidant enzyme activity, which resulted in cytoprotective effects in vitro, in vivo, and ex vivo patient cell material.^18,19,56^ Another important protective mechanism is related to its cellular metabolization under oxygen consumption, which leads to transcriptional activation and stabilization of hypoxia-inducible factor 1 alpha (HIF1α) both in vivo and in Caco-2 cells.^57−59^ Yin et al.^60^ demonstrated the importance of HIF1α activation for butyrate-mediated barrier reinforcement using a Caco-2 HIF1α knock-down model, while Hirota et al.^61^ evidenced its significance in Caco-2 resilience against *Clostridium difficile* toxin by blocking HIF1α.

In contrast to the reduced cytotoxicity, butyrate treatment enhanced the IL8 release in Caco-2 monocultures exposed to pretreated _fg_TiO_2_. This observation was particularly surprising as butyrate alone inhibited IL8 release in activated Caco-2 cells, which suggests that butyrate’s effect on IL8 may be dependent on the active cellular processes at the time of exposure. Again, already butyrate preincubation was sufficient to induce this effect. Although butyrate is typically associated with anti-inflammatory functions,^22,55^ studies demonstrated that this bias needs to be questioned. In a study on human tissue samples from inflammatory bowel disease patients, Magnusson et al.^62^ found strongly contrasting effects in diseased compared to healthy control tissue regarding the butyrate-induced anti-inflammatory immune regulation. This was suggested to be a reason for the often-established absence of positive effects of dietary butyrate on chronic inflammatory conditions of the intestine. Furthermore, Mizuno et al.^63^ investigated the effects of SFCA on different autoimmune inflammatory conditions where they observed that butyrate exposure ameliorated symptoms in some while exacerbating symptoms in other conditions. Even though their outcomes differed from the here-presented results, Fusunyan et al.^53^ also suggested a priming effect of butyrate on intestinal epithelial cells, making them susceptible to stressors like LPS and cytokines. A similar mechanism may have been involved here: as pretreated _fg_TiO_2_ alone also induced IL8 release, albeit at lower and nonsignificant levels, the dual exposure may have caused synergies that potentiated the individual effects. Synergistic effects have been described for butyrate and hyperosmolality,^64^ hyperthermia,^22^ and cytokines.^65^ Altogether, the addition of butyrate affects the responses of the cellular systems. Its specific functions, however, appear to be dependent on the ongoing cellular processes.

In the triple cultures, neither pretreated _fg_TiO_2_, butyrate alone, nor the combination of both induced consistent effects in any of the investigated end points regardless of the health status of the model. Butyrate-induced effects contrast between undifferentiated and differentiated Caco-2 cells,^22^ which Mariadason et al.^6^ suggested to be caused by an insufficient intracellular concentration of butyrate in differentiated cells. However, increasing the butyrate concentration was not a viable alternative as it caused significant adverse effects in multiple end points of both the stable and inflamed models. Caco-2 cells were isolated from a colon carcinoma and in an undifferentiated state represent colonocyte-like cells. As previously mentioned, butyrate is an important energy source and is readily metabolized by colonocytes.^66^ When grown to confluence, Caco-2 cells spontaneously differentiate and express enterocyte-like features.^67^ While enterocytes can utilize butyrate, they rather rely on glucose and glutamate for energy, both of which are present in our culture medium.^66^ It is, therefore, possible that the metabolization/utilization of butyrate also differs depending on the differentiation status, leading to different effects in undifferentiated versus differentiated Caco-2 cells.

Nevertheless, other studies reported butyrate-associated effects, especially in relation to inflammation. Using intestinal biopsies from ulcerative colitis patients and healthy controls, Magnusson et al.^62^ demonstrated a reduction in TNFα and IL10 in inflamed but not healthy tissue samples treated with butyrate. In an LPS-stimulated Caco-2/peripheral blood mononuclear cells (PBMC) coculture model, Korsten et al.^68^ observed a dose-dependent reduction in TNFα, IL10, and IL1β in the presence of butyrate. Also, Chen et al.^17^ demonstrated an inhibitory effect of butyrate on LPS-induced inflammation in a coculture of Caco-2 and RAW246.7 macrophages. Notably, the Caco-2 cells were only semidifferentiated after a culture period of 11 days, and rather than an apical treatment of the transwell, Chen and colleagues preincubated the macrophages in the basolateral compartment with 5 mM butyrate for 1 h before exposure to LPS. A rescuing effect by butyrate on intestinal barrier integrity has been reported both in vivo^17^ and in vitro^68^ albeit at higher concentrations of 5 and 8 mM, respectively. While Vancamelbeke et al.^69^ observed an overall beneficial effect on barrier integrity for butyrate in primary intestinal cell cultures of healthy and diseased tissues, it turned detrimental when coincubated with TNFα and IFNγ. Both cytokines are present in our inflamed model^41^ and may have inhibited butyrate’s functionality. Apart from the concentration itself, Korsten et al.^68^ suggested the type and magnitude of the immune reaction to be a determining factor for butyrate-induced effects. As the inflamed-like triple culture model mimics an active inflammation, including high cytokine release and cytotoxicity, the processes might have superimposed any beneficial effects of butyrate.

Our results on _fg_TiO_2_-exposed triple cultures contrast the increasing reports on TiO_2_-induced effects in diseased and susceptible models. TiO_2_ and _fg_TiO_2_ were reported to induce inflammation and preneoplastic lesions in healthy animals,^29,70^ adenoma formation and goblet cell count including expression of Muc1, Muc2, and Muc5AC,^71^ while more subtle effects, e.g., on antioxidant protein levels, or no effects were reported by others.^37,72^ In models of intestinal inflammation, TiO_2_ and _fg_TiO_2_ induced cell infiltration, hyperplasia, enhanced mucus density, aggravated the inflammatory condition, and delayed the healing process.^32,73,74^ Also, in vitro effects of _fg_TiO_2_ on differentiated intestinal cells and complex models were reported by others. Cao et al.^75^ observed cytotoxicity and ROS generation induced by lower concentrations of digested _fg_TiO_2_ than used here in an intestinal triple culture M-cell model combining Caco-2, HT29-MTX, and Raji B cells. Dorier et al.^35^ found pristine _fg_TiO_2_ to affect several tight junction proteins while no change in TEER was detected, with induction of a pro-inflammatory response, enhanced secretion of acidic mucus, and reduced MUC1 expression in Caco-2/HT29-MTX cocultures. In contrast, no effect on mucin glycosylation was detected.^33^

While it was not the aim of this study to elucidate the differences between pristine and pretreated _fg_TiO_2_, we nevertheless offer some suggestions for the observed cytotoxicity and induction of IL8 release by pretreated but not pristine _fg_TiO_2_. Various studies have investigated the impact of pretreatments mimicking the gastrointestinal passage both on the material used here^37,39^ and _fg_TiO_2_ from other sources.^76−78^ For all materials and simulant fluids tested, these studies unanimously reported a high degree of stability. Di Cristo et al.^39^ demonstrated that digestive simulant fluids did not affect the primary particle size, morphology, or crystallinity. Ferraris et al.^76^ furthermore reported stability in lysosomal environments, which renders extra- or intracellular ionic Ti unlikely to be responsible for the effect. _fg_TiO_2_ was previously found to induce adverse effects through ROS and oxidative stress.^34,47^ The intrinsic ability to generate ^•^OH radicals in acellular environments demonstrated by Proquin et al.^47^ was not supported by our results on either pristine or pretreated _fg_TiO_2_. The oxidant generating capacity of TiO_2_ may depend on the specifically investigated material, as also indicated from earlier observations with five different TiO_2_ ENM.^40^ However, minimalistic simulant fluids without enzymes, as used here, caused agglomeration/aggregation of _fg_TiO_2_, which persists throughout the intestinal phase (shown here and by Ferraris et al.^76^ and Cao et al.^75^). This might affect the particles’ sedimentation behavior and could therefore have affected the delivered dose rate. Whether this could explain the observed differences in effects between pristine and pretreated _fg_TiO_2_ remains to be investigated, in particular, because of contrasting findings regarding this phenomenon for other types of (nano)particles.^79,80^

## 5 Conclusions

We have demonstrated differential effects of butyrate in simple and complex in vitro models of the intestine that could affect the toxicity and pro-inflammatory capacity of pretreated _fg_TiO_2_. The cellular impact of butyrate was, however, relevant only in proliferating monocultures of undifferentiated Caco-2 cells and did not show relevance in advanced models. Our observations add to the question of the ideal in vitro testing and exposure strategy for the hazard assessment of particulate materials.^81^ In particular, the effect on cell cultures should be considered in the context of increasing complex pretreatment strategies of ENM, which will likely be translated to the testing of advanced materials. While we still consider the complex model used in this study to be a relevant tool for advanced hazard testing, its application will likely remain reserved for addressing highly specific questions concerning individual materials rather than screening larger numbers of materials. With this in mind, enhancing the applicability of simplistic models, such as proliferating Caco-2 monocultures with the use of physiologically relevant factors, might be a promising approach. Even though the data suggest that incorporation of butyrate, and potentially other SCFA, might be a relevant factor to enhance the physiological relevance of simple in vitro models, further research is needed to investigate the nature of the coexposure-induced effects.

## Supporting Information Available

Materials and methods, establishing _fg_TiO_2_ exposure concentrations, particle size distribution measured by DLS, effects of butyrate on cell viability in Caco-2 monocultures, effects of 24 h incubation with 10 and 20 mM butyrate in stable and inflamed triple cultures, cytokine release in stable and inflamed triple cultures after 24 h exposure to 10 or 20 mM butyrate, hydroxyl radical formation capacity, metabolic activity in Caco-2 cells after 24 h exposure to PVP-coated silver or ZnO ENM, EC_50_ of pristine and pre-treated Ag-PVP and ZnO ENM in Caco-2 cells, effects of butyrate on IL8 release in Caco-2 monoculture and on barrier integrity and expression of mucins and IL8, cytokine release in supernatants after triple culture in the presence or absence of butyrate, ZO-1 staining of epithelial transwell culture, example images of alkaline comet assay samples, and gene expression of DNA damage repair and oxidative stress markers (PDF)

## Abbreviations

_fg_TiO_2_: food-grade titanium dioxide (E171)
butyrate pre: 24 h preincubation with butyrate before exposure
butyrate co: coexposure of butyrate and another stressor
butyrate pre/co: 24 h preincubation with butyrate before subsequent 24 h coexposure of butyrate and another stressor
