# Supplementary material for: Effect of Butyrate on Food-Grade Titanium Dioxide Toxicity in Different Intestinal In Vitro Models
Source: Chem Res Toxicol. 2024 Aug 30;37(9):1501–14. doi: 10.1021/acs.chemrestox.4c00086 (PMC11409378; doi:10.1021/acs.chemrestox.4c00086)
Supplement: Supplementary file 1 — tx4c00086_si_001.pdf [file tx4c00086_si_001.pdf]

Supplementary Information

Effect of butyrate on food-grade titanium dioxide toxicity in different intestinal *in vitro* models

Janine M. Becht[†,‡], *Hendrik Kohlleppel*[†], *Roel P. F. Schins*[†], *Angela A. M. Kämpfer*[†]*

[†]IUF – Leibniz Research Institute for Environmental Medicine, Düsseldorf, Germany

[‡]current affiliation**:** Faculty of Biology, Technical University of Darmstadt, Germany

*Corresponding author contact details
Angela Kämpfer
Angela .Kaempfer@IUF-Duesseldorf.de
(+49)211 3389 351

Section 1 Materials & Methods

Establishing ${}_{fg}TiO_2$ exposure concentrations: In its 2021 Safety Assessment of Titanium Dioxide, the European Food Safety Authority (EFSA) has included exposure estimates for various population subgroups, ranging from infants to the elderly[1]. Focusing on the refined exposure assessment for an adult (70 kg bodyweight) with a daily ${}_{fg}TiO_2$ intake between 0.6 and 5.5 mg/kg bw/day, one obtains a mean daily exposure of 42-385 mg/day. Assuming 100% of the ingested ${}_{fg}TiO_2$ passes the oesophagus, stomach and duodenum, and a total surface area of 32 m^2 in the small intestine (based on the revised assessment by Helander and Fändriks (2014)[2], a concentration of 0.001-0.012 mg ${}_{fg}TiO_2$ cm^{-2} would be achieved, if the whole ingested content was evenly distributed throughout the intestine. For several reasons, this is an unlikely scenario as ${}_{fg}TiO_2$

- is ingested over several occasions per day rather than once,
- is ingested within a food matrix and therefore reaching the intestine within a chyme bolus
- and may be adhering to specific sites with higher affinity, e.g. inflamed lesions, as has previously been demonstrated both in human tissue and in a colitis mouse model[3, 4].

Therefore, the higher concentration of 80 µg cm^{-2} was used to represent hotspot exposure where accumulation of ${}_{fg}TiO_2$ particles occurred.

Section 2 Supplementary figures

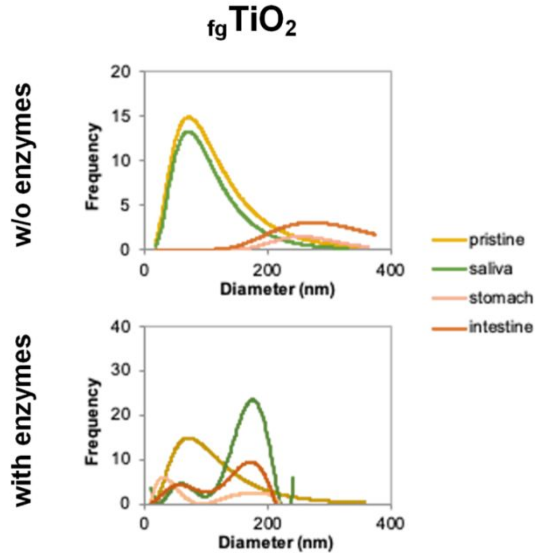

Figure S1 Particle size distribution measured by DLS. $_{fg}TiO_2$ was analysed in pristine form or following a cascaded incubation in fluids simulating the main phases of the human digestive process.

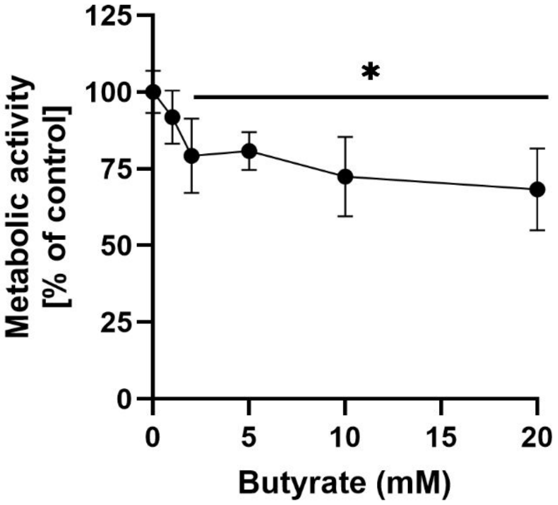

Figure S2 Effects of butyrate on cell viability in Caco-2 monocultures. Cells were exposed to 0-20 mM butyrate for 24 h before the metabolic activity was quantified by WST-1 assay. (mean ± SD, N=3; *p≤0.05 compared to corresponding control by one-way ANOVA and Dunnett's post hoc test)

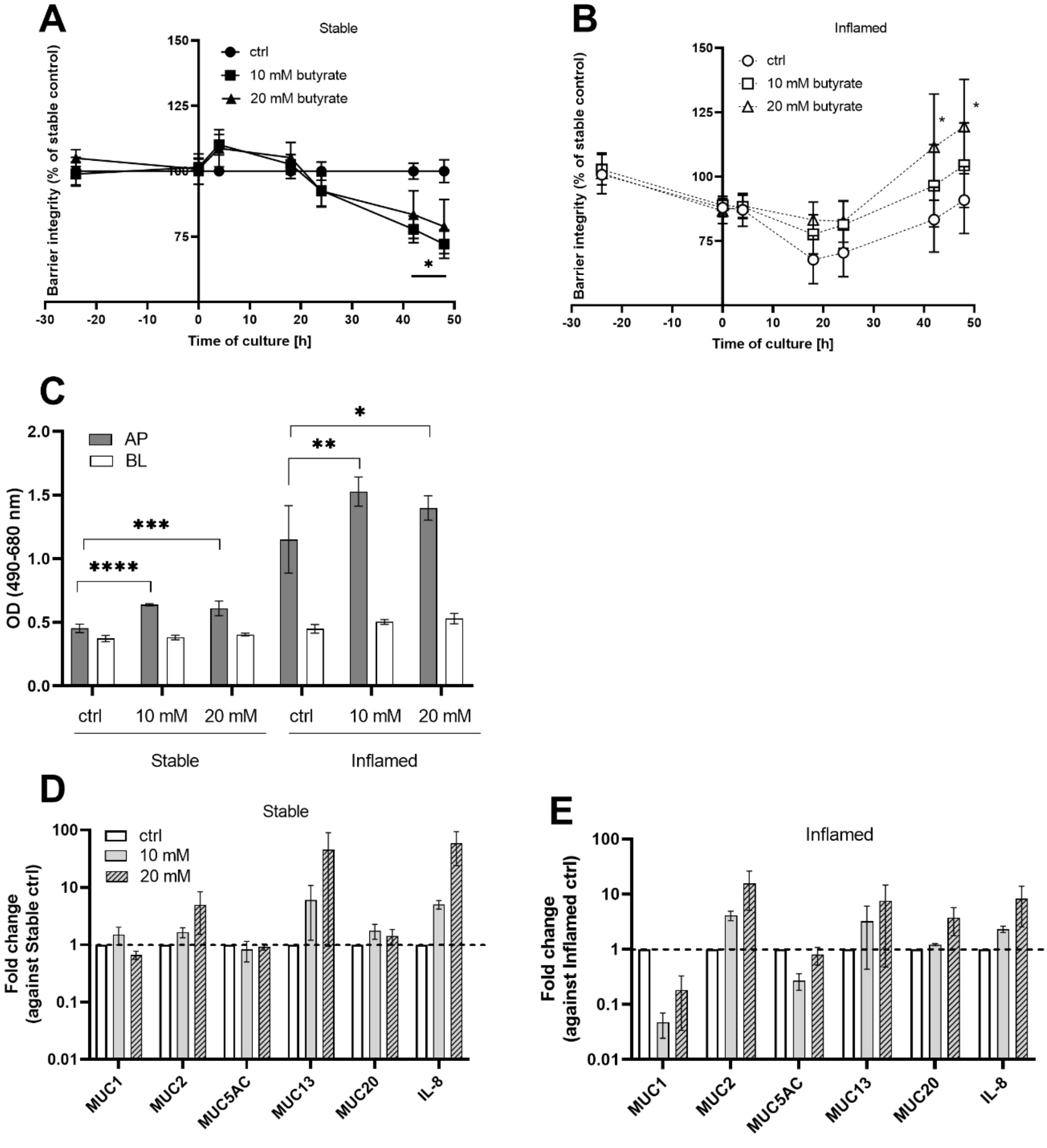

Figure S3 Effects of 24 h incubation with 10 and 20 mM butyrate in stable and inflamed triple cultures. (A,B) Barrier integrity, (C) LDH release in apical and basolateral supernatants after 48 h triple culture and 24 h butyrate exposure, (D,E) gene expression of mucins MUC1, MUC2, MUC5AC, MUC13 and MUC20 as well as IL8 in epithelial transwell cultures after 48 h stable or inflamed triple culture and 24 h exposure to butyrate (mean ± SD, A-C: N=3; D,E: N=2; *p≤0.05 / **p≤0.01 / ***p≤0.005 / ****p≤0.01 against corresponding control; AP: apical, BL: basolateral; ctrl = unexposed control conditions of the stable and inflamed model)

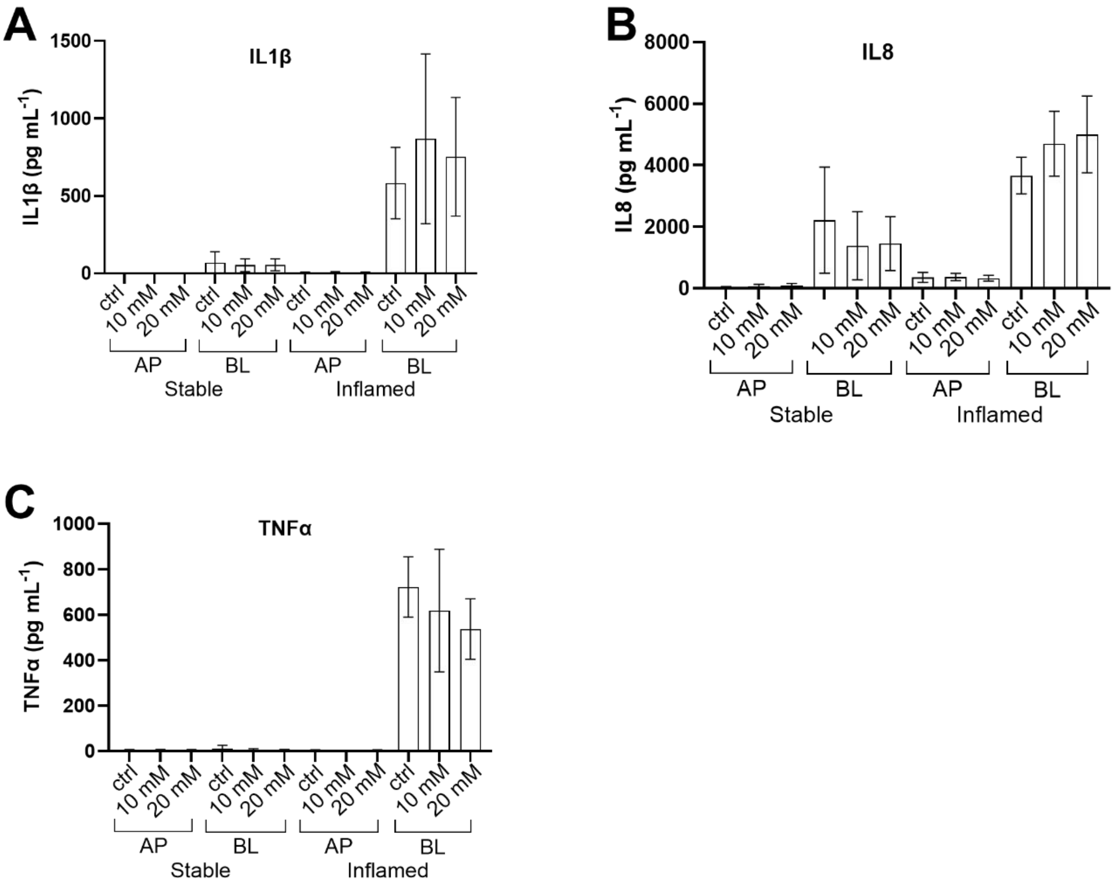

Figure S4 Cytokine release in stable and inflamed triple cultures after 24 h exposure to 10 or 20 mM butyrate. After 48 h stable or inflamed triple culture and 24 h exposure to 10 or 20 mM butyrate (A) IL1β, (B) IL8 and (C) TNFα were quantified in apical and basolateral supernatant. (mean ± SD, N=3; ctrl: unexposed control conditions of the stable and inflamed model)

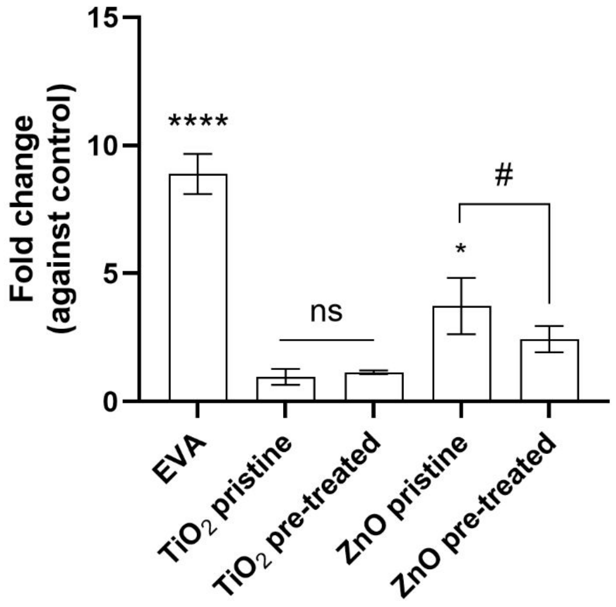

Figure S5 Hydroxyl radical formation capacity. EVA (fly ash) as positive control as well as $_{fg}TiO_2$ and ZnO in pristine or pre-treated state were mixed with hydrogen peroxide and the spin-trap DMPO. Electron paramagnetic resonance spectra were measured in three independent experiments. The amplitudes of the positive control and the samples relative to dH_2O were assessed as a measure of hydroxyl radical formation. (mean ± SD, N=3; *p≤0.05/****p≤0.001 compared to dH_2O control by one-way ANOVA, $^{\#}$p≤0.05 compared to pristine material by t-test)

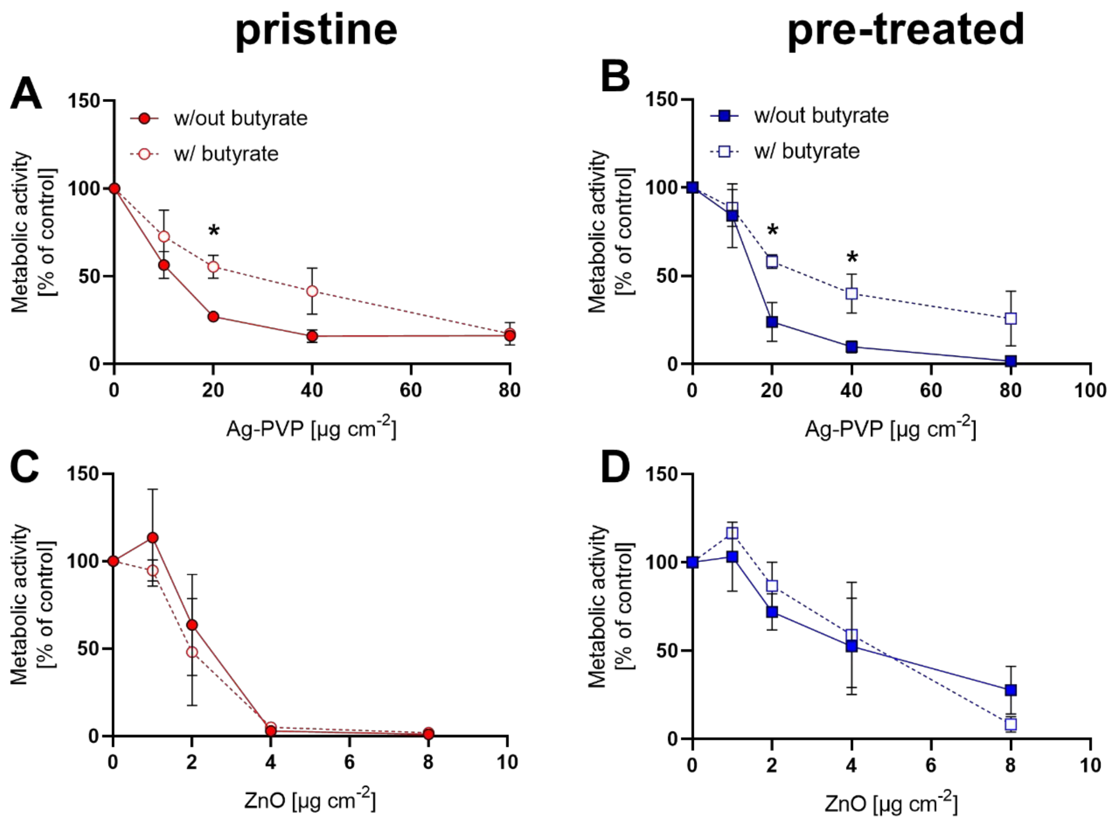

Figure S6 Metabolic activity in Caco-2 cells after 24 h exposure to (A,B) PVP-coated silver (Ag-PVP) or (C,D) zinc oxide (ZnO) engineered nanomaterials (ENM). Cells were exposed to pristine Ag-PVP and ZnO or following pre-treatment in absence or presence of 1 mM butyrate. After 24 h, the metabolic activity was measured by WST-1 assay and expressed against the corresponding control. (mean ± SD, N=3, *p≤0.05 compared to the corresponding exposure concentration without butyrate)

Table S1 EC_{50} (µg cm^{-2}) of pristine and pre-treated Ag-PVP and ZnO ENM in Caco-2 cells in absence or presence of 1 mM butyrate.

	**Pristine**		**Pre-treated**	
	**W/out butyrate**	**W/ butyrate**	**W/out butyrate**	**W/ butyrate**
Ag-PVP	11.40 ± 0.76	29.53 ± 7.93	14.69 ± 3.25	30.46 ± 6.64*
ZnO	2.26 ± 0.64	2.09 ± 0.62	4.46 ± 1.61	4.58 ± 1.41

*p≤0.05 compared to EC_{50} without butyrate by t-test (mean ± SD, N=3)

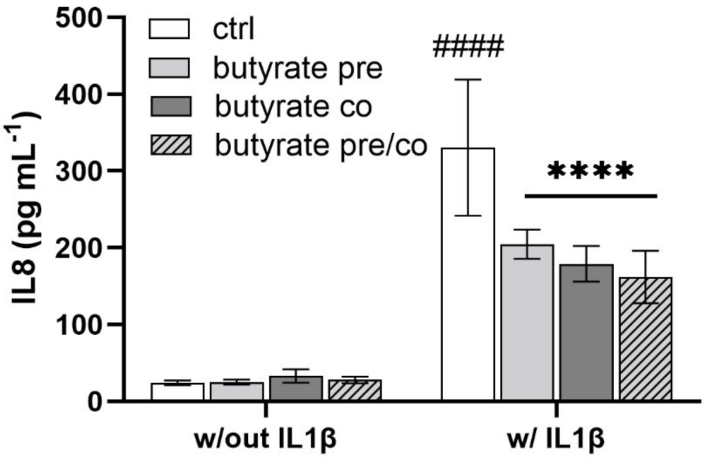

Figure S7 Effects of butyrate on IL8 release in Caco-2 monocultures. The effect of 1 mM butyrate on IL8 release was tested without or with activation of cells by 10 ng mL-1 IL1β. Different incubation regimes were tested: 24 h pre-incubation with butyrate, 24 h co-exposure with butyrate , and 24 h pre-incubation and 24 h co-exposure with butyrate. (mean ± SD, N=3; ####$p \leq 0.001$ compared to control without IL1β; *$p \leq 0.05$ / ****$p \leq 0.001$ compared to corresponding control by one-way ANOVA and Dunnett's post hoc test; ctrl: unexposed control without butyrate treatment)

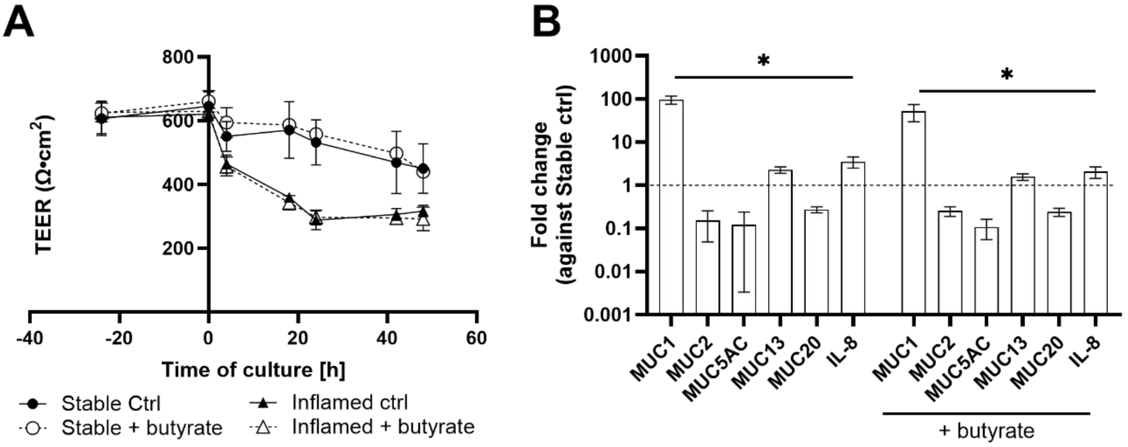

Figure S8 Effect of butyrate pre-incubation and co-exposure on barrier integrity and expression of mucins and IL8 in stable and inflamed triple cultures. (A) Stable and inflamed triple cultures were established (t_0) and cultured without butyrate (ctrl) or with 1 mM butyrate for 48 h. (B) After 48 h of stable and inflamed triple culture, the epithelial cells were analysed for the expression of mucins MUC1, MUC2, MUC5AC, MUC13 and MUC20 as well as IL8. The results were expressed as fold-change against the corresponding stable triple culture control. (mean ± SD, A: N=2, B: N≥3; *$p \leq 0.05$ compared to corresponding stable triple culture control by t-test; ctrl: unexposed control conditions of the stable and inflamed model)

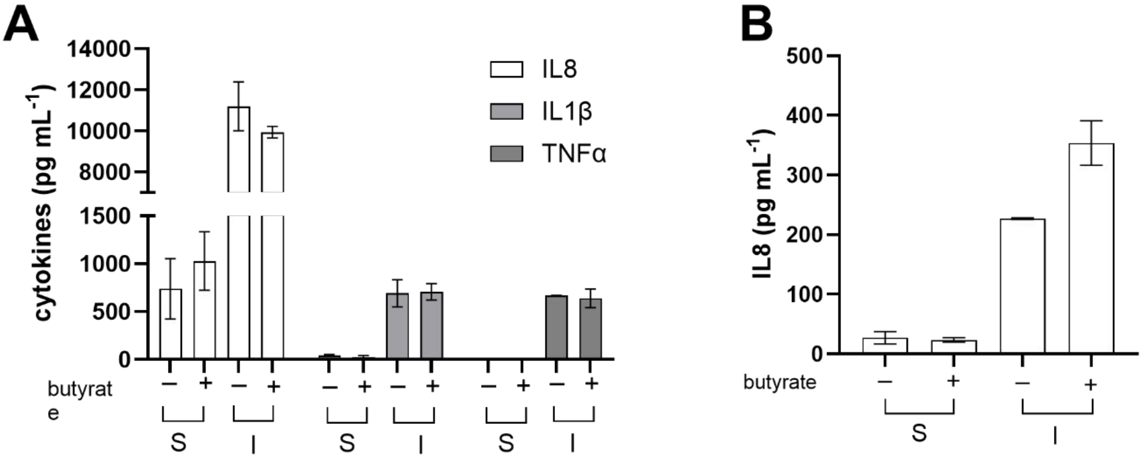

Figure S9 Cytokine release in (A) basolateral and (B) apical supernatants after 48 h of stable and inflamed triple culture in presence or absence of butyrate. Stable and inflamed triple cultures were established and maintained for 48 h without (-) or with (+) 1 mM butyrate. Apical supernatants were also analysed for IL1β and TNFα, however, both cytokines were below the detection limit in all conditions. (mean ± SD, N=2)

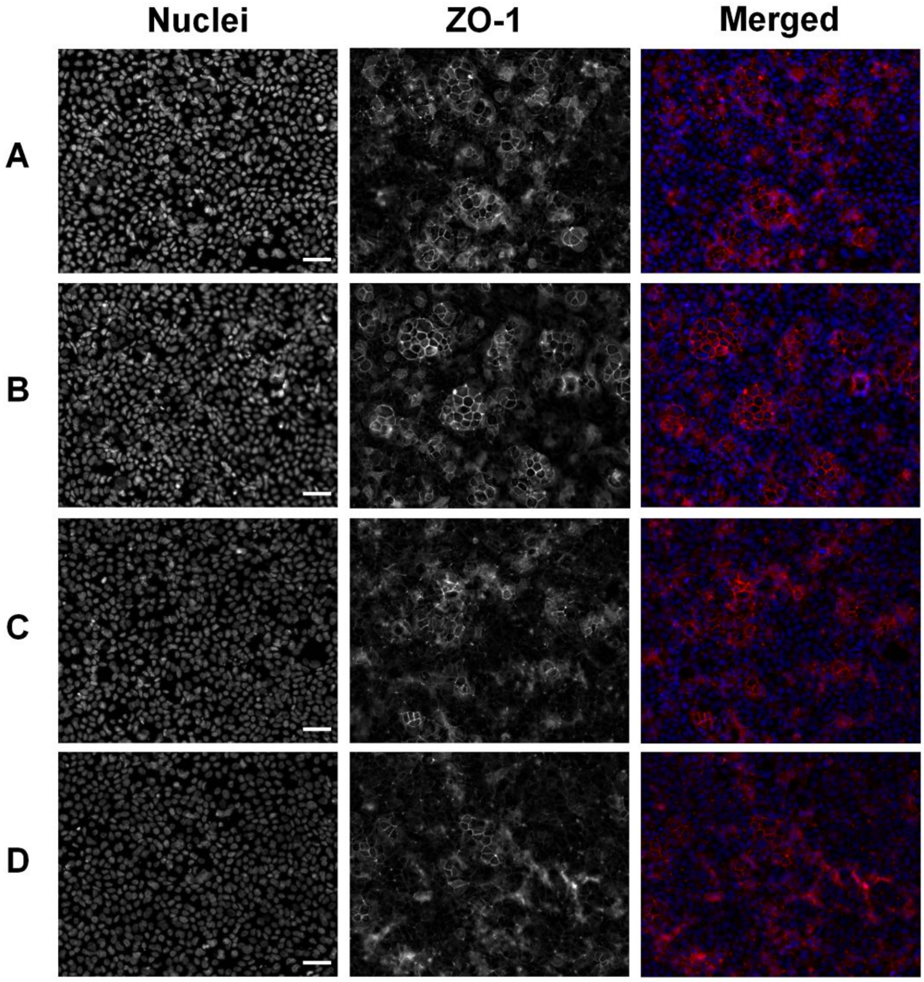

Figure S10 ZO-1 staining of epithelial transwell cultures after 48 stable (A,B) or inflamed (C,D) triple culture in absence (A,C) or presence (B,D) of butyrate. Nuclei were stained with DAPI. As Caco-2 and E12 cell morphology is highly different, imaging all cells in one plane is difficult to accomplish. The visible "cell islands" are clusters of E12 cells within the layer of Caco-2 cells. (10x magnification, scale bar = 50 µm).

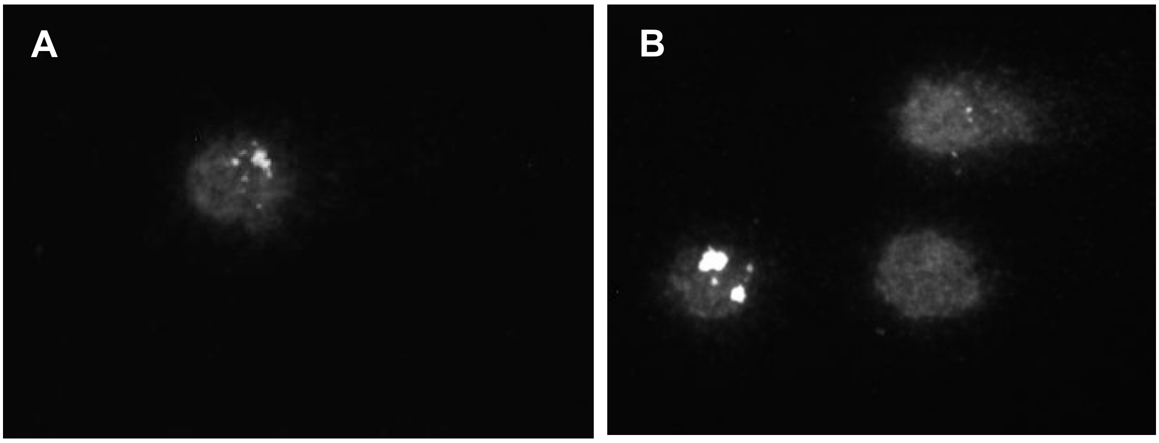

Figure S11 Example images of alkaline comet assay samples after 24 h exposure to 40 µg cm^{-2} pre-treated $_{fg}TiO_2$ in (A) absence or (B) presence of 1 mM butyrate

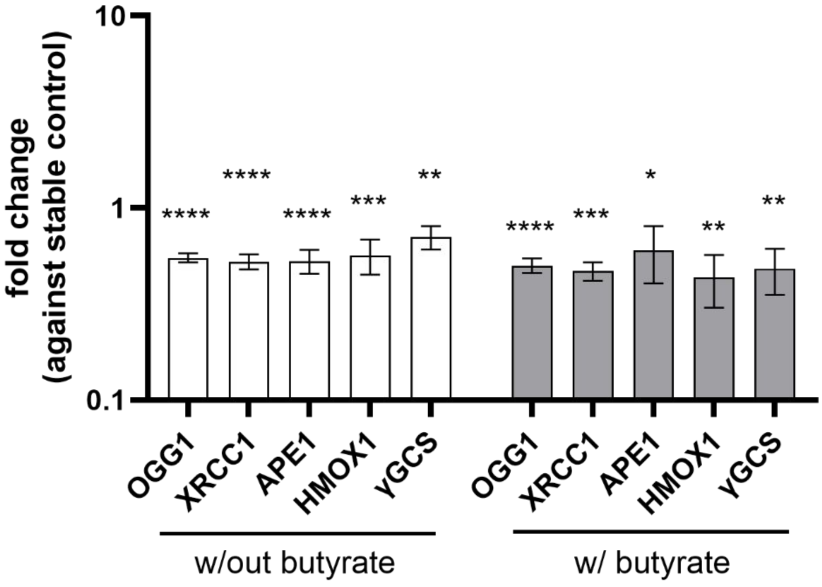

Figure S12 Gene expression of DNA damage repair and oxidative stress markers in the epithelial cells of inflamed triple cultures compared to the expression in the stable model. The results were normalized to the unexposed negative controls and β-Actin as reference gene. The depicted fold changes were derived from the ΔΔCT-values. (mean ± SD, w/out butyrate: N=4, w/ butyrate: N=3; */**/***/****p≤0.05/0.01/0.005/0.001 compared to the corresponding stable triple culture control by t-test)

References

(1) EFSA. Safety assessment of titanium dioxide (E171) as a food additive. *EFSA J* **2021**, *19* (5), e06585. DOI: 10.2903/j.efsa.2021.6585.

(2) Helander, H. F.; Fändriks, L. Surface area of the digestive tract – revisited. *Scand J Gastroenterol* **2014**, *49* (6), 681-689. DOI: 10.3109/00365521.2014.898326.

(3) Schmidt, C.; Lautenschlaeger, C.; Collnot, E.-M.; Schumann, M.; Bojarski, C.; Schulzke, J.-D.; Lehr, C.-M.; Stallmach, A. Nano- and microscaled particles for drug targeting to inflamed intestinal mucosa—A first in vivo study in human patients. *J Control Release* **2013**, *165* (2), 139-145. DOI: 10.1016/j.jconrel.2012.10.019.

(4) Watanabe, A.; Tanaka, H.; Sakurai, Y.; Tange, K.; Nakai, Y.; Ohkawara, T.; Takeda, H.; Harashima, H.; Akita, H. Effect of particle size on their accumulation in an inflammatory lesion in a dextran sulfate sodium (DSS)-induced colitis model. *Int J Pharm* **2016**, *509* (1-2), 118-122. DOI: 10.1016/j.ijpharm.2016.05.043.
